# COX-2 inhibition improves immunotherapy and is associated with decreased numbers of myeloid-derived suppressor cells in mesothelioma. Celecoxib influences MDSC function

**DOI:** 10.1186/1471-2407-10-464

**Published:** 2010-08-30

**Authors:** Joris D Veltman, Margaretha EH Lambers, Menno van Nimwegen, Rudi W Hendriks, Henk C Hoogsteden, Joachim GJV Aerts, Joost PJJ Hegmans

**Affiliations:** 1 Department of Pulmonary Medicine, Erasmus MC Rotterdam, The Netherlands

## Abstract

**Background:**

Myeloid-derived suppressor cells (MDSC) are a heterogeneous population of immature cells that accumulates in tumour-bearing hosts. These cells are induced by tumour-derived factors (e.g. prostaglandins) and have a critical role in immune suppression. MDSC suppress T and NK cell function via increased expression of arginase I and production of reactive oxygen species (ROS) and nitric oxide (NO). Immune suppression by MDSC was found to be one of the main factors for immunotherapy insufficiency. Here we investigate if the *in vivo *immunoregulatory function of MDSC can be reversed by inhibiting prostaglandin synthesis by specific COX-2 inhibition focussing on ROS production by MDSC subtypes. In addition, we determined if dietary celecoxib treatment leads to refinement of immunotherapeutic strategies.

**Methods:**

MDSC numbers and function were analysed during tumour progression in a murine model for mesothelioma. Mice were inoculated with mesothelioma tumour cells and treated with cyclooxygenase-2 (COX-2) inhibitor celecoxib, either as single agent or in combination with dendritic cell-based immunotherapy.

**Results:**

We found that large numbers of infiltrating MDSC co-localise with COX-2 expression in those areas where tumour growth takes place. Celecoxib reduced prostaglandin E2 levels *in vitro *and *in vivo*. Treatment of tumour-bearing mice with dietary celecoxib prevented the local and systemic expansion of all MDSC subtypes. The function of MDSC was impaired as was noticed by reduced levels of ROS and NO and reversal of T cell tolerance; resulting in refinement of immunotherapy.

**Conclusions:**

We conclude that celecoxib is a powerful tool to improve dendritic cell-based immunotherapy and is associated with a reduction in the numbers and suppressive function of MDSC. These data suggest that immunotherapy approaches benefit from simultaneously blocking cyclooxygenase-2 activity.

## Background

MDSC are a heterogeneous population of immature myeloid cells. These cells can inhibit anti-tumour responses in an antigen-specific and in a non-specific way [1,2]. Antigen-specific suppression takes place in lymphoid organs and depends on reactive oxygen species (ROS) production, leading to T cell tolerance. Antigen-non-specific suppression takes place at the tumour site and is mainly dependent on nitric oxide (NO) secretion, causing T cell specific apoptosis [2-4]. More recently, it has been shown that the heterogeneous group of MDSC can be subdivided into three major groups. Polymorph nuclei MDSC (PMN-MDSC), mononuclear MDSC (MO-MDSC) and Gr-1^low ^MDSC [5-7]. Greifenberg *et. al*. showed that the MO-MDSC and a subpopulation of Gr-1^low ^MDSC can inhibit T cell proliferation by the production of NO, in contrast to the PMN-MDSC that did not show this suppressive capacity [5]. However, the exact mechanisms that play a role in the induction of T cell tolerance by MDSC subtypes need to be further explored. As ROS production by MDSC also contributes to the induction of tolerance we determined the ROS producing capacity of these subpopulations of MDSC. In addition, we investigated if ROS production by these different MDSC populations can be influenced.

The production of ROS by MDSC is highly depending upon cyclooxygenase-2 (COX-2) enzyme activity [8,9]. The inducible COX-2 enzyme is essential in the biosynthesis of prostaglandins. Over-expression of COX-2 has been described as an important factor in tumour development. Therefore, high expression of COX-2 has been correlated with poor prognosis in cancer [10-12]. In addition, several studies showed the relevance of COX-2 inhibition in cancer progression [13-15]. Although the relation between COX-2 over-expression and prostaglandin E2 (PGE_2_) synthesis in cancer has been studied extensively, the impact on the tumour microenvironment is still under investigation [16-18].

Increased evidence indicates that immune suppressive cells, recruited by tumour-derived factors, are the main cause of failure of novel anti-cancer therapies, including immunotherapy [4,19-24]. We investigated dendritic cell (DC)-based immunotherapy in a murine model of mesothelioma. It was found that the efficacy of this treatment is hampered by the highly immunosuppressive environment in mesothelioma [25]. Adjacent to this, it was found that COX-2 over-expression in mesothelioma correlates with poor prognosis [26]. Combining the current knowledge on MDSC, COX-2 over-expression and immune suppression, we aimed to determine the effect of selective COX-2 inhibition on MDSC populations in the optimization of DC-based immunotherapy. We studied the effect of celecoxib on different MDSC populations in a murine model for mesothelioma. Since ROS is one of the principal factors leading to induction of T cell tolerance, we focused on the effects on ROS production by different MDSC populations during celecoxib-treatment.

## Methods

### Animals and cell lines

BALB/c mice (specific pathogen free [SPF], female, 6-8 weeks old) were purchased from Harlan (Zeist, The Netherlands) and were housed under pathogen-free conditions at the animal care facility of Erasmus MC. All experiments were approved by the local ethical committee for animal welfare (Erasmus University Committee of Animal Experts, Rotterdam, the Netherlands) and complied with the guidelines for the welfare of animals in experimental neoplasia by the United Kingdom Coordinating Committee on Cancer Research (UKCCCR), and by the Code of Practice of the Dutch Veterinarian Inspection. The mesothelioma AB1 cell line was kindly provided by Professor B.W.S. Robinson (School of Medicine and Pharmacology, Sir Charles Gairdner Hospital Unit, The University of Western Australia, Perth, Australia).

### Tumour growth of murine mesothelioma in BALB/c mice

BALB/c mice were divided into 4 groups. Each group consisted of 6 mice. On day 0, all mice were injected with a lethal dose of 0.5 × 10^6 ^AB1 tumour cells. From day 0 onwards, group 1 and 3 received a control diet while group 2 and 4 received celecoxib diet (500 mg celecoxib/kg [Celebrex^®^; Pfizer, New York, NY, USA]). Both were starch-based diets manufactured by Harlan Teklad (Madison, WI, USA) as described by T. Hahn *et al*. [27]. Mice in group 3 and 4 received DC-based immunotherapy consisting of 1 × 10^6 ^tumour-lysate loaded dendritic cells at day 10.

Dendritic cells were culture from bone marrow using RPMI 1640 (Gibco, Carlsbad, CA, USA) supplemented with 5% heat inactivated fetal bovine serum (FBS [Hyclone, Waltham, MA, USA]), 20 ng/ml GM-CSF (kindly provided by Kris Thielemans, VU Brussels, Belgium), and 50 μM β-mercaptoethanol (Sigma-Aldrich BV, Zwijndrecht, The Netherlands) for 9 days. At day 8, cells were pulsed with tumour-lysate (to the equivalent of three AB1 cells per DC) and overnight matured with 100 ng/ml LPS E. Coli 026:136 (Sigma-Aldrich BV). On the day of vaccination, DCs were harvested and purified by Lympholyte-Mammal (Cedarlane, Hornby, ON, Canada) density gradient centrifugation, and washed three times in phosphate-buffered saline (PBS [Gibco]). Cells were resuspended at a concentration of 1 × 10^6 ^viable cells in 500 μl PBS and intraperitoneally injected.

The occurrence of tumour growth, body weight, physical well-being and survival were measured for 2 months, as described previously [28].

### Immunohistochemistry on tumour cells and biopsies

Tumour cells were cultured in RPMI supplemented with 5% FBS on two chambers Falcon culture slides (BD biosciences, Erebodegem, Belgium) starting with 5 × 10^4 ^AB1 cells per well. Celecoxib was added to the culture when cells reached a confluence of 60% for 24 hours in a concentration of 10 μg/ml and 100 μg/ml. Resected tumour material was obtained from the peritoneal cavity of AB1 inoculated BALB/c mice at day 15. Tumour biopsies were embedded in Tissue-Tek II optimum cutting temperature (OCT) medium (Miles, Naperville, IL, USA), snap-frozen in liquid nitrogen and stored at -80°C. Tissue sections (6 μm) were cut on a HM-560 cryostat (Microm, Heidelberg, Germany).

For the COX-2 expression a rabbit affinity-purified IgG against murine COX-2 was used (Cayman chemical, Montigny-le-Bretonneux, France) (this antibody shows no cross-reactivity with COX-1). For the COX-1 expression a rabbit affinity-purified IgG against murine COX-1 was used. These primary antibodies were incubated for 1 hour at room temperature. Binding of antibodies was detected using the goat anti-rabbit alkaline phosphatase (AP) (Sigma-Aldrich). Naphtol-AS-MX-phosphate (0.30 mg/ml [Sigma-Aldrich]) and new fuchsine (160 mg/ml in 2 M HCl [Chroma-Gesellschaft, Köngen, Germany]) were used as substrate. Hematoxylene was used as counterstaining. The specificity of the antibodies was checked using a protein concentration-matched non-relevant monoclonal antibody and PBS.

Mouse mesothelioma biopsies were double stained for Gr-1-FITC (clone RB6.8C5) and COX-2. As secondary antibodies horseradish peroxidase (HRP) conjugated goat anti-FITC (Rockland, Gilbertsville, PA, USA) and AP-conjugated goat anti-rabbit (Sigma-Aldrich) were used. Naphtol-AS-MX-phosphate and 1 mM Fast Blue (Sigma-Aldrich) were used as substrate for AP and NovaRed was used as substrate for HRP, according to the manufacturer's instructions (Vector, Burlingame, CA, USA).

### Flow cytometry

Spleens were aseptically removed, and mechanically dispersed in cold HBSS (Invitrogen). Cell suspensions were filtered through a 100 μm nylon cell strainer (BD Biosciences), depleted of erythrocytes by osmotic lysis, washed twice in RPMI medium containing 5% FBS, and adjusted to a concentration of 1 × 10^6 ^cells/ml in FACs-buffer.

Splenocytes were stained with the following optimally diluted mAbs: Ly6c (FITC conjugated), MHCII (PE conjugated), CD11b (PercP-Cy5.5 conjugated) (all BD bioscience), CD8 (FITC conjugated), F4/80 (FITC conjugated), CD4 (PE conjugated), CD31 (PE-Cy7 conjugated), B220 (Alexa fluor 700 conjugated), Ly6g (APC-Cy7 conjugated) (all eBioscience), CD11c (PE-Texas red conjugated [Caltag, Burlingame, CA, USA]), and a live/dead marker (DAPI [Invitrogen]). Splenocytes were restimulated in the presence of GolgiStop (BD biosciences) for 4 hours using anti-CD3 and intracellular stained for Granzyme B (PE conjugated [Caltag]) and IFN-γ (APC conjugated [BD Biosciences]). Viability was determined by live dead aqua (Invitrogen). Acquisition of eight to nine colour samples was performed on a FACs LSR II cytometer (BD Biosciences). The analysis and graphical output were performed using FlowJo software (Tree Star Inc., Costa Mesa, CA, USA).

### ELISA

PGE_2 _levels in the peritoneal washes were determined using a specific ELISA assay for PGE_2 _(R&D systems, Abingdon, UK). Manufacturer's recommended protocols were followed. Serum was diluted appropriately to ensure that readings were within the limits of accurate detection.

### Nitric oxide and its reactive products (NO)

Equal volumes of peritoneal wash (150 μl) were mixed with Greiss reagent (1% sulfanilamide in 5% phosphoric acid and 0.1% *N*-1-naphthyl-ethylenediamine dihydrochloride in double-distilled water). After 30 min incubation at room temperature, the absorbance was measured at 548 nm using a microplate reader (Bio-Rad). Nitrite concentrations were determined by comparing the absorbance values for the test samples to a standard curve generated by serial dilution of a stock solution of sodium nitrite.

### Reactive oxygen species (ROS)

The oxidation-sensitive dye dichlorodihydrofluorescein diacetate (DCFDA, Sigma-Aldrich) was used for the measurement of ROS production by splenocytes. Cells were incubated at 37°C in DMEM (Invitrogen) in the presence of 1 μM DCFDA for 60 min, and washed twice with cold PBS. Cells were stained with Abs directed against Gr-1 (PE conjugated [BD biosciences]) and CD11b (PercP-Cy5.5 conjugated [BD biosciences]) as previously described. Cells were washed with cold PBS after 20 min incubation. Acquisition of the samples was performed on a flowcytometer.

### Tumour-specific lysis assay

Spleens were aseptically removed, and mechanically dispersed in cold PBS. Cell suspensions were filtered through a 100 μm nylon cell strainer (BD Biosciences), depleted of erythrocytes by osmotic lysis, washed twice in RPMI, and adjusted to a concentration of 4 × 10^6 ^cells/ml in RPMI medium supplemented with 5% FBS. After 48 h of culture, spleen cells were washed extensively. Mouse AB1 cells were incubated with 100 μCi of Na_2 _^51^CrO_4 _(ICN Biomedicals) for 2 hours at 37°C, washed three times, resuspended in culture medium at a concentration of 5 × 10^4 ^cells/ml. Splenocytes (150.000 cells per well) from either naïve mice, untreated mice, or mice treated with DC-based immunotherapy were mixed with 5 × 10^3 ^radiolabeled AB1 target cells and added to wells of a 96-well round-bottom microtiter plate (0.2 ml final volume) to achieve the desired effector:target (E:T) ratios. To determine the suppressive capacity of splenocytes from mice treated with control diet or celecoxib diet, 150.000 splenocytes from DC treated mice were mixed with 150.000 splenocytes from mice treated with either control diet or celecoxib diet. After 24 hour incubation, cells were mixed with radiolabeled AB1 target cells and added to wells of a 96-well round-bottom microtiter plate (0.2 ml final volume) to achieve the desired effector:target (E:T) ratios.

Plates were incubated for 4 hours at 37°C in a humidified atmosphere containing 5% CO_2_, and cell-free supernatants were collected from each well. The amount of ^51^Cr released from lysed AB1 target cells was determined by γ scintillation counting. Percent lysis was calculated using the formula: corrected % lysis = 100×(experimental release - spontaneous release [target cells incubated in medium alone])/(maximum release[2% Triton X-100 as lysing agent]-spontaneous release).

### Statistical analysis

Data are expressed as mean ± SD. Comparisons between groups were made using the t-test. A two-tailed p-value < 0.05 was considered significant. Data presented as a percentage of tumour-free animals were analysed with Kaplan-Meier survival-curves, using the log-rank test to determine significance.

## Results

### Identification of myeloid-derived suppressor cell subsets

Splenocytes from mice that were inoculated with a lethal dose of AB1 tumour cells and placed on a control or celecoxib diet were stained for flowcytometric analysis.

**Figure 1:**
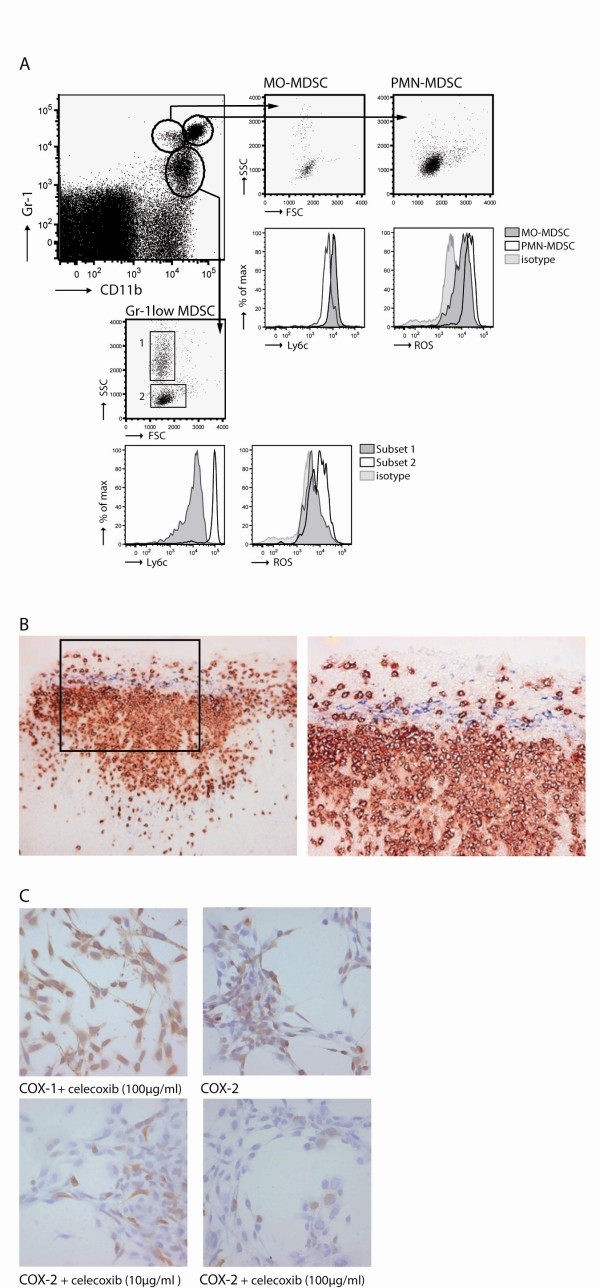
**Identification of MDSC and myeloid cell types in tumour-bearing mice**. A) PMN-MDSC expressed high levels of Gr-1, CD11b. MO-MDSC have a slightly higher expression of Ly6c. A third population was identified showing a low expression of Gr-1. Based on FSC/SSC this population was further subdivided into two subsets; SSC^low ^expressing high Ly6c and SSC^high ^expressing low Ly6c. ROS production was measured for each subtype. B) Histology on tumour sections showed Gr-1^+ ^(red) at the rim of the tumour in close contact with COX-2 expressing cells (blue). [Magnification: 200× (left) and 400× (right)]. C) Inhibition of COX-2 activity was tested *in vitro *by adding celecoxib to AB1 cell cultures. AB1 cells were cultured for 24 hours with medium, 10 μg/ml, or 100 μg/ml celecoxib and stained with anti-COX-2 and anti-COX-1 (as control) antibodies.

Similar to the most recent published data, three populations of Gr-1^+^CD11b^+ ^cells were identified [5-7]. The Gr-1^low ^MDSC could be further subdivided in two populations based on size and inner complexity of the cells (forward [FSC]/side scatter [SSC]). The physical characteristics combined with Ly6c expression was used to visualize other intrinsic differences between the different groups. We found that Ly6c expression was lower on the Gr-1^low ^MDSC with a high SSC (subset 1) in contrast to the Gr-1^low ^MDSC with low SSC (subset 2). Ly6c expression on PMN-MDSC was lower then Ly6c expression on MO-MDSC (Figure 1A).

MDSC can induce T cell tolerance by producing ROS. ROS production was highest in PMN-MDSC followed by MO-MDSC. Subset 2 of the Gr-1^low ^MDSC had the capability to produce ROS, whereas the majority of the cells in subset 1 were not able to produce ROS (Figure 1A).

Tumour biopsies were obtained and stained for the presence of Gr-1^+^CD11b^+ ^cells. Large areas with infiltrating positive cells were found at the rim of the tumour. Since COX-2 is an essential enzyme for catalyzing the biosynthesis of tumour-derived prostaglandin E2 (PGE_2_), a molecule that induces MDSC [7], biopsies were stained for COX-2 expression. COX-2 expressing cells were found at the border of the tumour closely located near areas with Gr-1^+^CD11b^+ ^infiltrating cells (Figure 1B).

Next we investigated if the enzyme activity of tumour-derived COX-2 could be inhibited by celecoxib *in vitro*. Therefore, cultured AB1 tumour cells were incubated with different dosages of celecoxib. After 24 hours supernatant was removed and cells were stained for the expression of COX-2 and COX-1 as a control. COX-1 expression was not effected by the selective COX-2 inhibitor celecoxib. COX-2 expression was reduced in a doses-depended manner (Figure 1C).

In conclusion, these data show that MDSC in the spleens of tumour-bearing mice can be subdivided into three groups. Additionally, we demonstrate that PMN-MDSC and MO-MDSC produce high ROS. The group of Gr-1^low ^MDSC could be further subdivided into two subsets based on FSC/SSC, Ly6c and ROS production. Large areas with infiltrating Gr-1^+^CD11b^+ ^cells were found at the rim of the tumour in close contact to COX-2 expressing cells. COX-2 expression by AB1 tumour cells was decreased by celecoxib *in vitro *in a dose-depended manner.

### Reduction of MDSC by dietary administration of celecoxib

We investigated the effect of celecoxib treatment on the four MDSC subsets that were identified in the spleen of tumour-bearing mice. Splenocytes from mice that were inoculated with AB1 tumour cells and received celecoxib diet or control diet were analyzed for the presence of the MDSC subsets.

**Figure 2:**
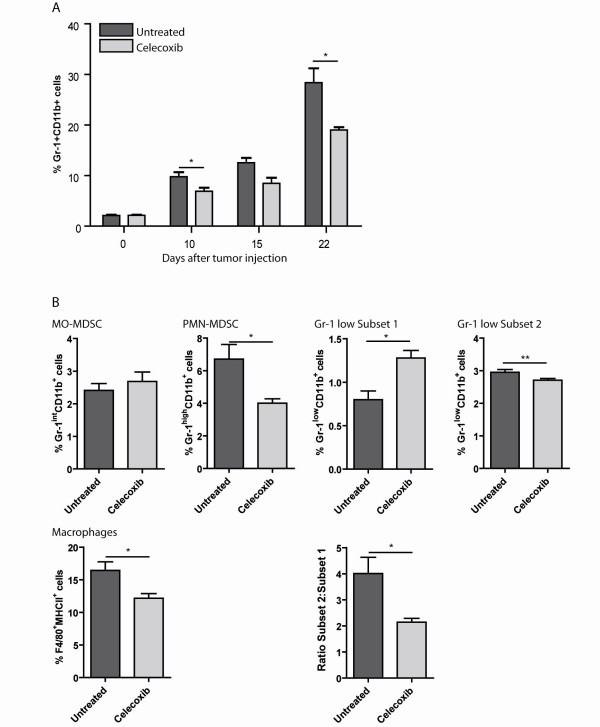
**Reduction of MDSC in tumour-bearing mice after celecoxib-treatment**. A) Accumulation of MDSC (characterized by the expression of Gr-1 and CD11b) in tumour-bearing mice receiving control diet or celecoxib diet: day 0 n = 4/4, day 10 n = 5/5, day 15 n = 3/2, day 22 n = 3/3). A significant difference was found at day 10 and day 22. * p = 0.034, ** p = 0.031. B) Splenocytes from mice receiving control diet or celecoxib diet were analyzed 10 days after tumour injection (n = 5/5). Subtypes were identified as described in Figure 1. A significant reduction was found in PMN-MDSC (p = 0.021) and in subset 2 from the Gr-1^low ^MDSC population (p = 0.041) in mice receiving celecoxib diet. A significant increase was found in subset 1 from the Gr-1^low ^MDSC population (p = 0.007), leading to a shift in ratio (p = 0.020) in celecoxib treated mice. The percentage of F4/80^+^MHCII^+ ^cells was significantly reduced in mice receiving celecoxib diet (p = 0.022).

Ten days after tumour injection, the absolute number of MDSC was significantly lower in mice receiving celecoxib diet compared with mice receiving control diet. This difference was more pronounced at day 22 after tumour injection (Figure 2A). When MDSC subsets (MO-MDSC PMN-MDSC and the two subpopulations of Gr-1^low ^MDSC as described in Figure 1A), a significant decrease in the percentage of PMN-MDSC was found. In addition, a shift between subset 1 and subset 2 within the Gr-1^low ^MDSC was observed leading to an increase of subpopulation 1. The total numbers of Gr-1^low ^MDSC did not differ between celecoxib treated and untreated animals. More recently, it has been shown that macrophages can be derived from MDSC [6,29]. Therefore this cell population was also analyzed. Macrophages were characterized by FACS based on their FSC/SSC, in combination with the expression of CD11b, F4/80 and MHCII on their membrane. A significant reduction in macrophages was observed in celecoxib treated mice compared to untreated mice (Figure 2B).

To summarize, a significant decrease in the number of MDSC in the spleen was found at day 10 after tumour inoculation and became more diverged when tumour progressed in celecoxib treated mice compared with untreated animals. Furthermore, we found that the reduction in MDSC after dietary celecoxib treatment was mainly caused by a reduction of PMN-MDSC and a shift in subsets of the Gr-1^low ^MDSC.

### Reduced ROS production by MDSC subsets

We next investigated if dietary celecoxib treatment not only influenced the number of MDSC in the spleen but also affects the function of the MDSC subsets. It has been shown that MDSC can down regulated the ζ-chain on T cells by ROS production in the lymphoid organs and thereby induce T cell tolerance [19,30]. We analyzed splenocytes of dietary celecoxib treated and untreated mice for their capability to produce ROS.

**Figure 3:**
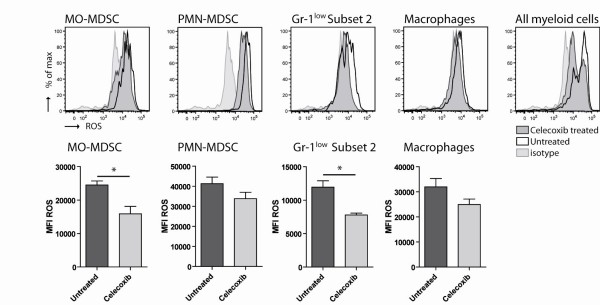
**Decreased ROS production by MDSC and myeloid cell types after celecoxib-treatment**. Production of reactive oxygen species (ROS) by (immature) myeloid subtypes from the spleen of tumour-bearing mice was measured. Splenocytes were obtained from tumour-bearing mice receiving either control diet or celecoxib diet for 10 days after tumour injection. Subtypes were identified based on characteristics as described in Figure 1. ROS production was reduced by MDSC in tumour-bearing mice treated with celecoxib diet. Mean fluorescent intensity (MFI) of ROS was significantly lower in MO-MDSC (p = 0.015) and Gr-1^low ^Subset 2 MDSC (p = 0.017) after celecoxib-treatment. The MFI of ROS on macrophages did not significantly change (p = 0.14).

Celecoxib treatment reduced ROS production in all MDSC subsets, especially in the Gr-1^low ^MDSC subset 2 and MO-MDSC. There was a trend toward a reduction of ROS production by macrophages, though this reduction was not significant (p = 0.14). Analyzing ROS production by all myeloid cells revealed that ROS production was decreased in celecoxib treated mice compared to untreated animals (Figure 3).

In summary, ROS production was most pronounced in PMN-MDSC. Dietary celecoxib treatment reduced ROS production in all MDSC subtypes but is most effective in the MO-MDSC and Gr-1^low ^MDSC subset 2 both in percentage as well as the median fluorescence intensity (MFI).

### Reduction of COX-2 expression in tumour tissue after dietary celecoxib treatment

After we observed that celecoxib treatment reduced the number and function of MDSC in the spleen of tumour-bearing mice we investigated if dietary treatment of mice with celecoxib also affects MDSC in the tumour environment. Therefore tumour biopsies were analyzed for the expression of COX-2 enzyme and the presence of infiltrating MDSC. In addition, the peritoneal cavity of tumour-bearing mice was washed with PBS and analyzed for tumour-derived PGE_2 _and NO production.

**Figure 4:**
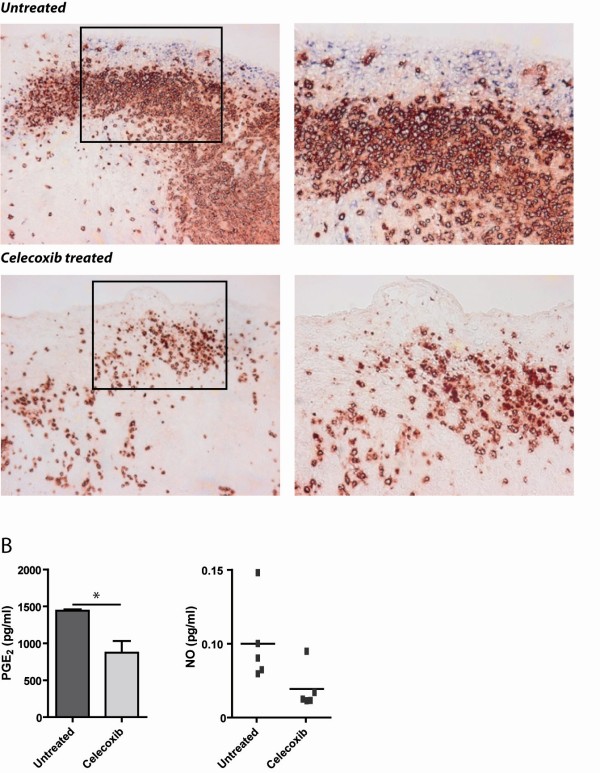
**Reduction of COX-2 expression after celecoxib-treatment**. A) Tumour sections of untreated (n = 7) and celecoxib treated mice (n = 8) were immunohistochemically double-stained for the presence of Gr-1 (red) and COX-2 expression (blue). No expression of COX-2 was found in tumour sections from mice with dietary celecoxib. Gr-1^+ ^cells were reduced in tumour sections of mice treated with celecoxib [magnification 200× left and 400× right]. B) PGE_2 _levels were measured in peritoneal washings of untreated (n = 7) versus celecoxib treated mice (n = 8) (p = 0.0061). NO was detected using Greiss reagent.

Tumour was infiltrated with massive amounts of Gr-1^+^CD11b^+ ^cells, mainly at the border of the tumour. These cells were in close contact to COX-2 expressing tumour cells. Treatment with celecoxib showed that the Gr-1^+^CD11b^+ ^cells were more restricted and that COX-2 expression at the rim of the tumour was lost (Figure 4A).

Additionally, tumour-derived PGE_2 _was decreased in the peritoneal wash of mice receiving celecoxib diet compared to animals on control diet. Also a reduction in NO concentrations was found (Figure 4B).

These data show that treating mice with celecoxib decreased the expression of COX-2 and thereby reduced MDSC both systemically as well as in the local microenvironment of the tumour. This reduced COX-2 expression is accompanied by with a reduction in PGE_2 _and NO levels. Moreover, the infiltration of Gr-1^+^CD11b^+ ^cells in tumour areas was reduced by celecoxib-treatment.

### Reduction of immune suppression after celecoxib treatment

In our previous study we have shown that DC-based immunotherapy in this murine model leads to the induction of a strong anti-tumour response [28]. However, the effectiveness of the anti-tumour response was negatively influenced when tumours sizes increased [28]. It has been addressed that one of the main reasons for immunotherapy failure is the induction of T cell tolerance by immune suppressive cells. To determine if treatment with celecoxib also intercepts the induction of T cell tolerance the following experiments were performed. A chromium release assay was performed to investigate the effects on tumour specific lysis in different conditions.

**Figure 5:**
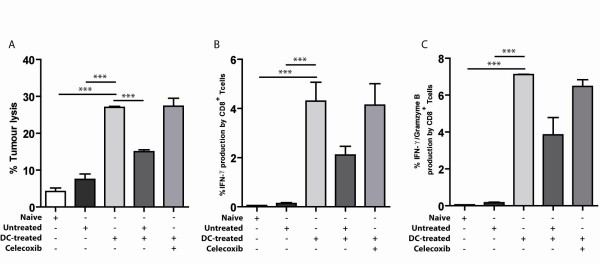
**Improved T cell function after celecoxib-treatment**. A) The amount of ^51^Cr released from lysed radiolabeled AB1 target cells was determined to compare cytolytic activity of splenocytes isolated from naïve mice (n = 4) tumour-bearing mice (n = 5) and DC-treated mice (n = 4). To determine the inhibitory effect of MDSC in the spleen, splenocytes from mice receiving control diet or celecoxib diet were co-cultured with splenocytes of DC-treated mice (the number of DC-treated splenocytes was equal in all conditions 150.000 cells/well). Percentage of lysis was calculated using the formula: corrected % lysis = 100 × (experimental release - spontaneous release [target cell incubated in medium alone])/(maximum release - spontaneous release). Splenocytes from DC-treated mice were significantly better capable of lysing tumour cells compared to naïve (p < 0.0001) and tumour-bearing mice (p < 0.0001). The addition of splenocytes from a tumour-bearing mice treated with control diet significantly reduced the lytic capacity of splenocytes from DC-treated mice (p = 0.0002) whereas the addition of splenocytes from tumour-bearing mice treated with celecoxib diet did not affect the lytic capacity of splenocytes from DC-treated mice (p = 0.887). B) Percentages of IFN-γ^+ ^CD8^+ ^cells within the spleen were determined by intracellular FACs staining. DC-treatment significantly improved IFN-γ production by CD8^+ ^cells compared to naïve mice (p < 0.0001) and tumour-bearing mice (p = 0.001). Conditions as described above. C) IFN-γ/Granzyme B production by CD8^+ ^cells was measured by intracellular FACs staining revealing a significant difference between naïve (p < 0.0001) and tumour-bearing mice(p < 0.0001) compared to CD8^+ ^cells in the spleen of DC-treated mice.

There was no specific lysis of tumour cells when splenocytes of naïve mice or tumour-bearing mice were co-cultured with radioactive labelled tumour cells. When splenocytes from mice treated with DC-based immunotherapy were co-cultured with tumour cells a massive induction of tumour lysis was observed, demonstrating that DC-based immunotherapy induces tumour specific recognition by immune cells. To examine the suppressive effect of MDSC in the spleen, splenocytes from DC-treated mice were co-cultured with splenocytes of tumour-bearing mice treated with either control diet or celecoxib diet. After 4 hour incubation at 37°C, the splenocyte mixtures were co-cultured with the radioactive labelled tumour cells. Although the total amounts of splenocytes were equal in all conditions, a significant reduction in anti-tumour activity was found when splenocytes of control diet animals were added. In contrast, the addition of splenocytes from tumour-bearing mice treated with dietary celecoxib did not reduce the lytic capacity of splenocytes from mice treated with DC-immunotherapy (Figure 5A).

IFN-γ and granzyme B production by CD8^+ ^T cells showed similar results. CD8^+ ^T cells from the spleen of naïve and tumour-bearing mice were not capable of IFN-γ and granzyme B production. Production of IFN-γ and granzyme B was significantly increased after DC-treatment. Co-culture of splenocytes from DC-treated mice with splenocytes from tumour-bearing mice treated with control diet or celecoxib diet, showed that CD8^+ ^cells were affected by splenocytes of mice that had received the control diet while splenocytes of celecoxib treated mice did not affect the capability of CD8^+ ^cells to produce IFN-γ and granzyme B (Figure 5B).

In conclusion, these data show that anti-tumour responses induced by DC-treatment, are affected by suppressive cells in the spleen of tumour-bearing mice. However, the anti-tumour activity as indicated by AB1 lysis and IFN-γ/granzyme B production by CD8^+ ^T cells was no longer influenced when co-cultured with Splenocytes of mice receiving celecoxib diet, indicating that COX-2 inhibition leads to a reduction in suppressive immune cells.

### Dietary celecoxib improves DC-based immunotherapy

Next, we wanted to know if combining dietary celecoxib and DC-based immunotherapy would lead to an increased survival benefit. Previous studies showed that mice treated with DC-immunotherapy one day after tumour injection leads to 100% survival in DC-treated mice by inducing anti-tumour T cell activity. However, the efficacy of DC-treatment decreases with increasing tumour burden. To study the possible synergistic effect of celecoxib and immunotherapy a *suboptimal *DC-treatment protocol was used. Therefore in this protocol mice received tumour lysate-loaded DC 10 days after tumour injection. Mice receiving AB1 tumour cells developed signs of terminal illness after 12 days.

**Figure 6:**
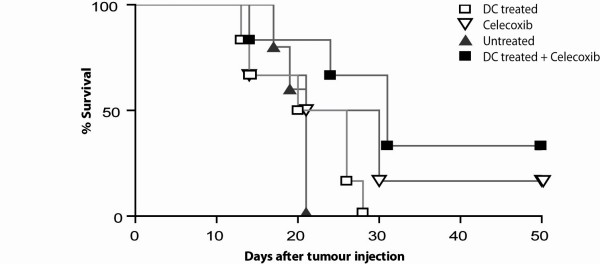
**Dietary celecoxib improves DC-based immunotherapy**. Mice were inoculated i.p. with 0.5 × 10^6 ^AB1 tumour cells at day 0. A *suboptimal *DC-treatment protocol was used. Mice were treated with DC-based immunotherapy at day 10 after tumour injection. Mice received control diet or celecoxib diet from day 1 onwards. All groups consisted of 6 mice. Survival was measured using Kaplan-Meier survival analysis. Combining DC-based immunotherapy with dietary celecoxib improved survival (p = 0.027), compared to a single treatment with celecoxib (p = 0.305) or DC-based immunotherapy (p = 0.456). All compared to no treatment.

Treatment with celecoxib alone prolonged survival to some extent; however this prolongation was not significant. No side effects were observed in mice receiving the celecoxib diet. The combination of dietary celecoxib and DC-immunotherapy led to a significant improvement of the immunotherapy (p = 0.038). In addition, combined treatment compared to no treatment significantly improved survival (p = 0.027) were single treatment with celecoxib or suboptimal DC-treatment did not improve survival (p = 0.305 and p = 0.455) (Figure 6).

This experiment showed that the combination of dietary celecoxib and immunotherapy is superior to single treatment in mice with high tumour burden.

## Discussion

We show that dietary administration of celecoxib leads to a significant decrease in the number and suppressive function of immature myeloid cells in the spleens and tumours of tumour-bearing mice. Subdivision of the heterogeneous group of immature myeloid cells revealed that three types could be identified in tumour-bearing mice, reflecting findings done by others [5,7]. The PMN-MDSC population was most frequently present in the spleen of tumour-bearing mice. This population was also most capable of ROS production. Previous studies showed that ROS production by MDSC is the main factor for inducing T cell tolerance by down regulation of the ζ-chain on activated T cells [19,30]. Celecoxib-treatment especially influenced this PMN-MDSC population compared to other MDSC subtypes in the spleen of tumour-bearing mice. Greifenberg *et. al*. recently showed that this population was not capable of NO production and therefore showed no suppressive effect on T cell proliferation in a T cell mixed leukocyte reaction (MLR) [5]. However, our data provides evidence that PMN-MDSC do play a significant role in tumour-induced immune suppression, since PMN-MDSC are most capable of producing ROS and thereby contribute to the induction of tolerance. Induction of tolerance can influence novel treatment strategies like DC-based immunotherapy negatively. Others have shown that immune suppression in the spleen in mainly dependent on ROS production whereas immune suppression in the tumour area is predominately depending on NO production by myeloid cells indicating that the increased PMN-MDSC population suppresses the anti-tumour T cell responses by producing ROS.

The Gr-1^low ^MDSC population can be divided into two subsets. We observed a shift in the Gr-1^low ^MDSC population of celecoxib treated mice leading to less ROS producing cells in this fraction. Greifenberg *et al*. showed that FSC/SSC low subset had suppressive capacity and were able to produce NO after LPS/IFN-γ restimulation. Next to this, we were able to show that this subset is also capable of ROS production whereas the FSC^high ^fraction within the Gr-1^low ^MDSC could not. Furthermore, we showed that celecoxib-treatment not only decreased the amount of immature myeloid cells in tumour-bearing mice, but that treatment also impaired ROS production by the different subpopulations of MDSC. Given the fact that the number of MDSC is reduced and the production of ROS by these cells is diminished makes them less capable of inducing T cell tolerance. The fact that T cell tolerance was no longer impaired after celecoxib-treatment was confirmed in a cytotoxicity assay showing that AB1 lysis was hampered when cells were co-cultured with splenocytes from mice who had received control diet while tumour specific lysis occurred when co-culture was performed with splenocytes from tumour-bearing mice treated with celecoxib diet.

Histological analyses revealed that COX-2 expression was mainly present in those areas where Gr-1^+^CD11b^+ ^cells were found. Almost all AB1 tumour cells express the COX-2 enzyme in culture. An explanation for this finding could be that tumour cells secrete high levels of PGE_2 _in order to attract MDSC. The other option is that COX-2 expression in culture is necessary for proliferation. However, by adding celecoxib to tumour cells the expression of COX-2 was reduced without affecting the metabolic activity of the cells (data not shown). *In vivo *COX-2 expressing tumour cells were present at the rim of the tumour tissue co-localizing with the Gr-1^+^CD11b^+ ^areas. COX-2 expression is diminished in tumour when mice were treated with celecoxib. The decrease in COX-2 enzyme expression has been observed in other studies [31-33]. The mechanism by which celecoxib perturbs COX-2 protein expression is not known. It has been suggested that PGE_2 _functions as a feedback on COX-2 protein expression and that celecoxib inhibits this loop [34]. It is also possible that the inhibitory effect of celecoxib on the NFκB pathway results in a reduced production of COX-2 proteins [35]. The reduction in COX-2 expression was accompanied by a reduction of PGE_2 _levels in peritoneal wash of celecoxib treated mice. Unfortunately no clear division can be made between the MDSC types in tumour tissue. Since Gr-1 (Ly6g) and Lyc6 are both expressed on all subtypes, no histological subdivision can be made using these markers. New histological markers are necessary for better classification of the different MDSC subtypes in tumour tissue.

Currently, we are investigating DC-immunotherapy in mesothelioma patients. Although we were able to induce an immune response in 4 out of 6 patients, the induced anti-tumour responses are opposed by immune suppressive cells like MDSC. Ochoa and colleagues have suggested that prostaglandin E2 produced by tumour cells induced the arginase I expression in MDSC [36]. For this reason, COX-2 inhibition to reduce MDSC in number and function has been proposed as promising strategy to improve immunotherapy. We have previously shown that upon injection of antigen-loaded DCs an effective immune response can be induced in a mouse model for mesothelioma depending on the timing of the immunotherapy [28]. In this study we were able to show that the reduction of MDSC *in vivo *was associated with an improved anti-tumour response and resulted in prolongation of survival in combination with DC-based immunotherapy. Same results were reported by others [37]. We were not able to verify that the improvement of the anti-tumour response in tumour-bearing mice treated with celecoxib was directly caused by the reduction in MDSC. Because MDSC are depending on cytokines, chemokines, prostaglandins and growth factors produced by surrounding cells for their function, we choose to mix total splenocytes instead of sorted Gr-1^+^CD11b^+ ^cells. For example MDSC are critically depending on IFN-γ production by T cells [2]. To determine that the abolishment of suppression in celecoxib treatment mice was most likely caused by the reduction of MDSC number and function, we screened splenocytes of all mice for other cell-types. No significant differences in the number of B cell, T cell (including Tregs and γδT cells) or macrophages between untreated and celecoxib treated tumour-bearing mice were observed. The number and function of MDSC did differ between the two groups (as described on in the result section). Therefore we conclude that the improved anti-tumour response is most likely caused be the reduction in MDSC number and function, though no firm conclusions on direct relations can be made.

We determined the effect of celecoxib treatment on MDSC; however, since COX-2 (and PGE_2_) is known to contribute to variety of cellular processes it is difficult to determine the solitary effect of a COX-2 inhibitor on MDSC *in vivo*. The role of COX-2 expression and tumour-derived PGE_2 _in cancer has been studied intensively. Significant correlations between the levels of COX-2 expression and survival were observed in many human cancers [38-40], including malignant pleural mesothelioma. COX-2 over-expression was found in the majority of mesothelioma (73% epithelial mesothelioma, 50% of mix-variants and 37% sarcomatoid mesothelioma). Survival analysis revealed that over-expression of COX-2 was related to worse prognosis. For this reason COX-2 inhibition has been proposed as potential therapeutic target for mesothelioma [10,11]. Also PGE_2 _contributes to proliferation, survival, angiogenesis, migration and invasion of tumour cells [41].

A rapid induction of MDSC was found during tumourigenisis in our mouse mesothelioma model. Although it is generally accepted that MDSC are part of the tumour microenvironment [19], their numbers or function may differ in various tumour cell-lines or other tumour model. This is caused by differences in the cytokine, chemokine and growth factor production of tumour cells that determines the specific microenvironments [42,43]. To determine which patients may benefit from celecoxib treatment alone or in combination with immunotherapy, more research is needed.

## Conclusions

Large numbers of infiltrating MDSC co-localise with COX-2 expression in tumour biopsies. Selective COX-2 inhibition by celecoxib reduced prostaglandin E2 levels *in vitro *and *in vivo*. Treatment of tumour-bearing mice with dietary celecoxib prevented the local and systemic expansion of all MDSC subtypes and also their suppressive function was impaired. Combining celecoxib with DC-based immunotherapy demonstrated highly activated cytotoxic T lymphocytes with superior immunostimulatory potency and anti-tumour activity because of the reduced MDSC expansion. This leads to a significant benefit in overall survival.

We conclude that celecoxib is a powerful tool to reduce the numbers and suppressive function of MDSC, which was associated with a beneficial effect of dendritic cell-based immunotherapy. Future studies will demonstrate the effectiveness of celecoxib treatment combined with dendritic cell-based immunotherapy in a clinical setting for cancer patients.

## Competing interests

The authors declare that they have no competing interests.

## Authors' contributions

JV: acquisition and interpretation of data; writing the manuscript. ML: carried out animal studies. MvN: carried out flowcytometric analysis. RH: performed the statistical analysis and data analysis. HH: have given final approval to the manuscript submission, revised the manuscript. JA: have made substantial contributions to conception and design, data interpretation. JH: have made substantial contributions to conception and design, data interpretation, revised the manuscript. All authors read and approved the final manuscript.

## Authors' information

Authors have recently published a clinical trial in the American Journal of Respiratory and Critical Care Medicine on the use of dendritic cell-based immunotherapy in mesothelioma patients. We showed for the first time the safety and feasibility of tumor lysate-pulsed dendritic cells as therapeutic adjuvants in mesothelioma patients and found distinct immune responses and antitumor responses in these patients. Now we are focusing on refinement of this approach.

## Pre-publication history

The pre-publication history for this paper can be accessed here:

http://www.biomedcentral.com/1471-2407/10/464/prepub
